# Theoretical Model for a Pneumatic Nozzle–Cylindrical Flapper System

**DOI:** 10.3390/mi16101148

**Published:** 2025-10-10

**Authors:** Peimin Xu, Kazuaki Inaba, Toshiharu Kagawa

**Affiliations:** 1 Department of Transdisciplinary Science and Engineering, Institute of Science Tokyo, Tokyo 152-8550, Japan; xu.p.aa@m.titech.ac.jp; 2 Institute of Science Tokyo, Tokyo 102-0073, Japan; tkagawa0256@gmail.com

**Keywords:** high-speed rotation spindle with aerostatic bearings, nozzle flapper, pneumatic, machining

## Abstract

To increase semiconductor production yield and meet the growing global demand, air bearings offering higher processing speeds and reduced friction losses have been proposed as an ideal solution. However, due to the non-contact support characteristic of air bearings, challenges such as shaft displacement caused by processing resistance inevitably arise. As an engineering requirement, the shaft must restrict lateral deflection to within 30 μm under transverse force. In our previous research, a compensation system using a nozzle–flapper mechanism as a displacement sensor was proposed to address shaft displacement. The effectiveness of the nozzle–flapper system in measuring shaft displacement was validated at rotational speeds up to 20,000 rpm. Furthermore, the compensation system’s ability to maintain the shaft’s initial position under a 5 N external force was verified in related collaborative research. In this study, building upon prior work, we further analyze the system characteristics of the cylindrical nozzle–flapper. This includes modeling the geometric space formed by the specific shape of the cylindrical flapper and nozzle and proposing an airflow hypothesis based on this geometry. The hypothesis is incorporated into the theoretical model of a standard nozzle–flapper system, resulting in an optimized theoretical method applicable to cylindrical configurations. Experimental results validating the effectiveness of the proposed model are also presented.

## 1. Introduction

Semiconductors are the cornerstone of various technological advancements and play an indispensable role in modern electronics. With the rapid innovation and iteration of electronic products, the global demand for semiconductors continues to grow, making the enhancement of semiconductor production increasingly urgent. It is widely acknowledged that a processing spindle with a higher rotational speed can improve processing efficiency [1]. According to the research of Shi et al. [2,3,4], air bearings due to their low friction and high-speed characteristics are considered an ideal solution for improving semiconductor processing performance. However, the non-contact support nature of air bearings introduces challenges; specifically, the supported shaft may experience significant displacement when subjected to processing resistance [2]. As an engineering requirement, the lateral deflection of the shaft must be limited to within 30 μm under transverse loading. To address this issue, our previous research [5] proposed a compensation system that uses a nozzle–flapper mechanism as a displacement sensor. Experimental verification demonstrated that the nozzle–flapper system can accurately measure micrometer-level shaft displacement at rotational speeds up to 20,000 rpm. We further investigated the dynamic characteristics of the nozzle–cylindrical flapper system. Based on the experimental results, a theoretical solution was proposed to ensure the stable operation of the system under dynamic conditions. Moreover, the effectiveness and validation under the dynamic condition of the compensation system in maintaining the shaft’s initial position under a 5 N external force were verified in the context of related collaborative research [6].

**Figure 1:**
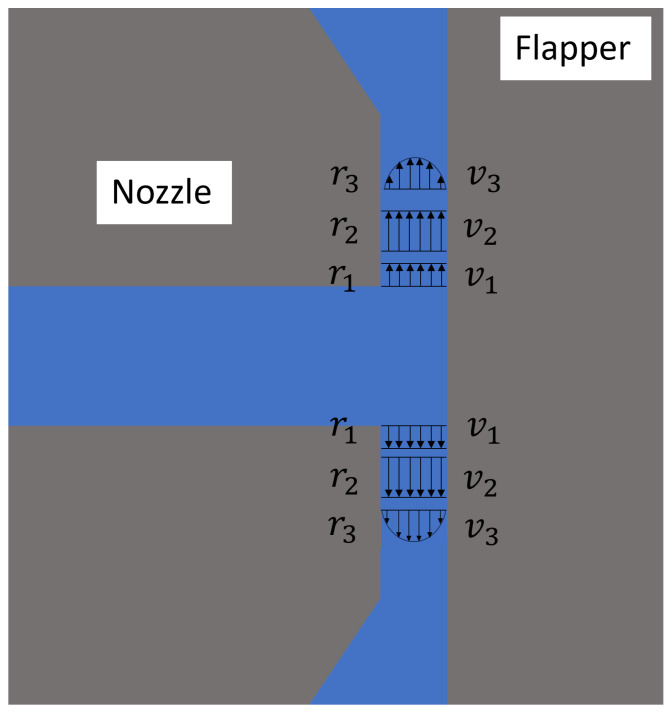
Geometric plane parallel to the axis of the cylindrical flapper (flat case).

**Figure 2:**
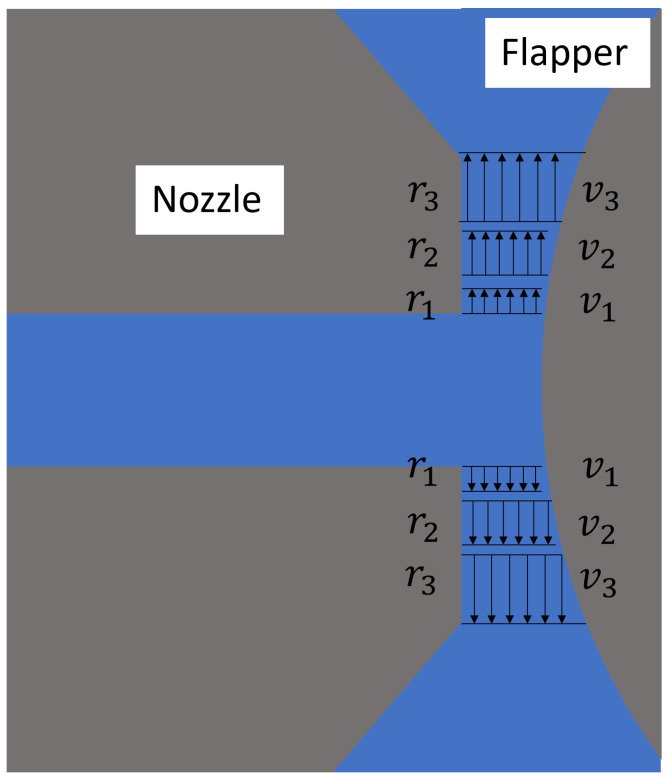
Geometric plane perpendicular to the axis of the cylindrical flapper (pure arc case).

**Figure 3:**
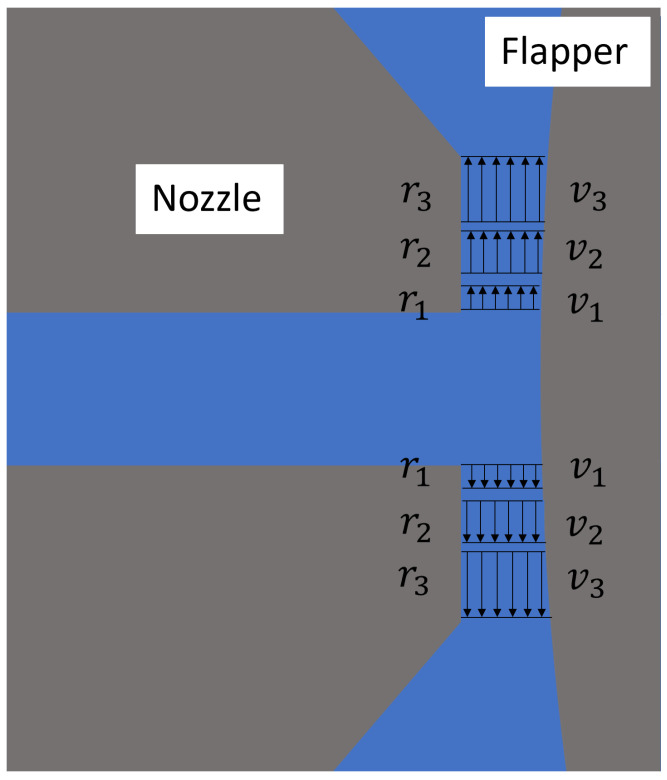
Geometric plane at 45 degrees to the axis of the cylindrical flapper (arc transition case).

Although the nozzle–flapper system, particularly the cylindrical type, has been validated for use in rotating body measurement, most previous studies have focused on traditional flat-type flapper systems, which are widely used as basic sensing components in pneumatic systems [7,8,9,10]. The unique geometric space formed between the cylindrical flapper and the nozzle significantly alters the actual air discharge characteristics. While research on hydraulic nozzle systems with non-flat geometries [11,12,13] may provide valuable insights, the fundamental difference in compressibility between water and air introduces notable variations in discharge behavior. The discharge characteristics of the nozzle–cylindrical flapper system partially resemble those of the flat-type system but also exhibit distinct behaviors, as illustrated in Figure 1, Figure 2 and Figure 3. The primary difference lies in the flow behavior between the nozzle and the flapper. As shown in Figure 1, the gap between the nozzle and the flapper remains constant during airflow, allowing for the application of the pressure depression theory [14,15] to model this behavior.

This configuration resembles that of a conventional flat-plate flapper, where the clearance between the nozzle and the flapper is uniform along the radial direction. Accordingly, the analysis can draw from studies on similar structures, such as annular orifice thrust bearings [16,17]. When air exits the nozzle and enters the clearance, it first undergoes an inertial acceleration stage (from $r_{1}$ to $r_{2}$ in Figure 1), converting a substantial portion of pressure energy into kinetic energy (from $v_{1}$ to $v_{2}$). During this process, a vena contracta forms as the airflow detaches from the nozzle wall, resulting in negligible axial velocity gradients across the same cross-section. Consequently, both wall friction and internal viscous resistance are minimal. Following this, the air enters the boundary layer development stage ($r_{2}$ to $r_{3}$ in Figure 1), during which it attaches to the surfaces of the nozzle and flapper, forming a boundary layer characterized by a velocity gradient distribution in the axial direction. This causes a decrease in the average velocity (from $v_{2}$ to $v_{3}$), and a slight recovery in radial pressure occurs more pronounced at larger clearance distances. Pressure recovery ceases once the boundary layer is fully developed. Subsequently, the flow enters the laminar flow stage, where pressure gradually decreases along the radial direction due to viscous resistance. This stage dominates discharge loss in the nozzle–flapper system and is indicated by a discharge coefficient of less than 1, as supported by the experimental results on thin-lip nozzles presented in [18]. In the case of a short nozzle edge, only the inertial acceleration stage is present, without boundary layer development or laminar flow, leading to a discharge coefficient close to 1. The above describes the complete airflow process through the clearance in this geometric plane. As previously noted, the influence of the inertial acceleration stage becomes more pronounced as the nozzle–flapper gap increases, causing the discharge coefficient to increase. However, due to the inevitable presence of the boundary layer and laminar flow stages, the discharge coefficient remains less than 1. In other words, this geometric plane exhibits greater discharge resistance than those at other angles, making it the most difficult path for air discharge.

It is important to emphasize that these flow characteristics are unique to the plane parallel to the axis of the cylindrical flapper. Consequently, the flow domain characteristics at other angles will differ from those of this geometric plane and can be further elucidated by analyzing the plane perpendicular to the cylindrical flapper axis. However, for the discharge characteristics illustrated in Figure 2 and Figure 3, the gap between the nozzle and the flapper tends to increase significantly during airflow. This variability makes it difficult to directly apply existing theoretical models. From the geometric structure of the nozzle–cylindrical flapper system, it can be observed that during the discharge process beginning with air exiting the nozzle, passing through the gap between the nozzle and the flapper, and finally flowing into the ambient environment, the majority of the flow cross-section resembles the configurations shown in Figure 2 and Figure 3. As a result, the overall discharge characteristics of the system cannot be directly modeled using existing theoretical approaches. Consequently, various empirical results and theoretical calculations developed for the flat-type nozzle–flapper system cannot be accurately applied to the static characteristic analysis of the nozzle–cylindrical flapper system in certain cases, resulting in calculation errors exceeding 15%.

The objective of this paper is to establish a theoretical calculation model capable of accurately describing the behavior of the nozzle–cylindrical flapper system. To achieve this, the three-dimensional geometry of the non-standard spatial gap between the nozzle and cylindrical flapper will first be characterized. Based on this spatial analysis, a hypothesis will be proposed to describe the air discharge flow tendency. Then, by applying momentum analysis to the control volume, integrating both the calculated spatial geometry and the flow tendency hypothesis, a compensation coefficient for the cylindrical flapper will be derived. Finally, this compensation coefficient will be used to optimize the traditional nozzle–flapper calculation model, and the validity of the improved model will be verified through the experimental results.

## 2. Discharge Characteristic Analysis of the Nozzle–Cylindrical Flapper System

Based on the above research, this section focuses on analyzing the discharge characteristics of the nozzle cylindrical flapper system. For a standard nozzle–flat plate flapper, the high geometric symmetry allows for simplification of the flow domain to a two-dimensional model. Along the radial direction of the nozzle, the clearance distance between the nozzle and the flapper remains constant. Therefore, in traditional nozzle–flapper analyses, the airflow at the nozzle edges is generally assumed to reach laminar flow [9]. This is the primary distinction between the flat plate and the cylindrical flapper configurations. To better understand the geometric peculiarities of the cylindrical flapper and their influence on airflow discharge, this paper compares two extreme geometric planes: the plane parallel to the cylindrical flapper axis (Figure 1) and the plane perpendicular to it (Figure 2), which are orthogonal to each other. Additionally, a neutral plane, located between these extremes, is also considered (Figure 3).

As shown in Figure 2, the most notable feature of the plane perpendicular to the cylindrical flapper axis is that the clearance distance between the nozzle and the flapper along the radial direction is no longer constant. From a flow cross-sectional perspective, the flow area along the radial direction now follows a cubic rather than a quadratic relationship. Flow domain analysis for this plane proceeds as follows: Initially, the air exits the nozzle and enters the clearance, separating from the wall and entering the inertial acceleration stage (from $r_{1}$ to $r_{2}$ in Figure 2), similar to the behavior in the plane parallel to the axis. However, due to the increasing clearance along the radial direction, the airflow, while remaining attached to the wall, cannot form a stable boundary layer and therefore cannot transition into laminar flow (from $r_{2}$ to $r_{3}$ in Figure 2). As a result, viscous resistance remains negligible throughout the process (as shown from $v_{2}$ to $v_{3}$ in Figure 2). The continuous inertial acceleration leads to a pronounced pressure gradient. From the standpoint of discharge resistance, this geometric plane offers the lowest resistance, resulting in the smoothest air discharge.

Considering the two geometric planes discussed above, the geometry at most other angular positions is expected to resemble that of the plane perpendicular to the flapper axis. In these configurations, the clearance distance between the nozzle and the flapper increases along the radial direction of the nozzle. As a representative case, this study analyzes a geometric plane positioned at a 45-degree angle to the cylindrical flapper axis, as shown in Figure 3. The distinguishing feature of this plane, in the perpendicular case, lies in the curvature of the flapper surface, which varies gradually along the radial direction.

Consequently, the clearance distance increases slowly at first (from $r_{1}$ to $r_{2}$ in Figure 3) and then accelerates more rapidly (from $r_{2}$ to $r_{3}$), producing a flow domain similar—yet slightly different—to that in the perpendicular case.

Given that most geometric planes exhibit similar discharge behavior, it is expected that the average discharge resistance in the cylindrical flapper system will be lower than that in the parallel plane (i.e., the flat flapper case). Therefore, under the same orifice diameter, nozzle diameter, and supply pressure, the static characteristics of the nozzle–cylindrical flapper—specifically, its sensitivity to control pressure changes—should exceed those of the conventional flat plate flapper.

## 3. Discharging Area Calculation and Momentum Analysis

**Figure 4:**
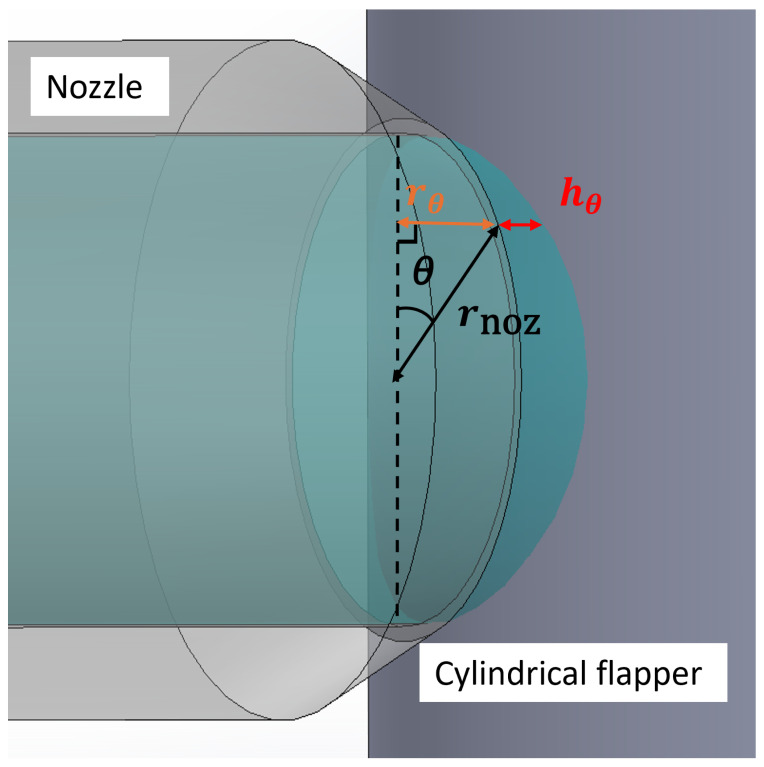
Schematic of discharge area (view oriented at 35 degrees to the side view).

From the above discussion, it can be concluded that the discharge characteristics of the cylindrical flapper differ from those of the traditional flat flapper. To model this difference, it is first necessary to calculate the air discharge area between the cylindrical flapper and the nozzle. As shown in Figure 4, by projecting the nozzle radius $r_{noz}$ onto the surface of the cylindrical flapper in the horizontal direction along the circumferential angle θ, the corresponding projection radius $r_{\theta }$ can be obtained. The relationship between these parameters is expressed as follows:

(1) $$\;r_{\theta }=r_{\mathrm{noz}}\cdot \sin\theta$$

**Figure 5:**
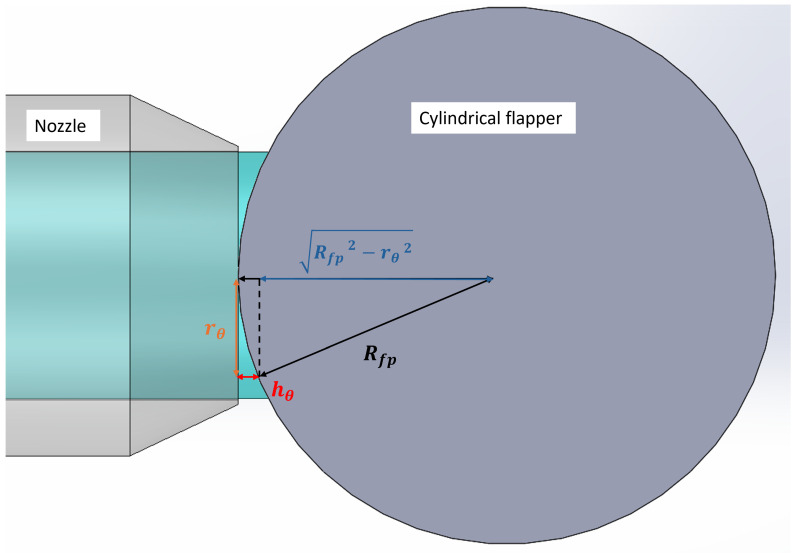
Schematic of discharge area (top view).

For each projection angle onto the cylindrical flapper, the corresponding $r_{\theta }$ can be used to calculate the perpendicular distance $h_{\theta }$ between $r_{\theta }$ and the arc of the cylindrical flapper surface (i.e., the straight-line distance from the edge of the nozzle aperture to the cylindrical flapper surface). As shown in Figure 5, the relationship among $h_{\theta }$ the flapper radius $R_{fp}$ and $r_{\theta }$ is given by the following:

(2) $$\;h_{\theta }=R_{fp}+x-\sqrt{{R_{fp}}^{2}-{r_{\theta }}^{2}}$$

In the projection perpendicular to the flapper’s axial direction, where $r_{\theta }$ = $r_{noz}$, the maximum perpendicular distance $h_{\max}$ is obtained. Its expression is as follows:

(3) $$\;h_{\max}=R_{fp}+x-\sqrt{{R_{fp}}^{2}-{r_{noz}}^{2}}$$

By integrating $h_{\theta }$ along the circumferential direction of the nozzle, the air discharge area $S_{fc}$ between the nozzle and the cylindrical flapper can be determined:

(4) $$\;S_{fc}=4\cdot \int_{0}^{\frac{\pi }{2}}h_{\theta }\cdot r_{noz}\cdot d\theta$$

When $R_{fp}$ > $r_{noz}$, this area integral can be approximated using an elliptical equation:

(5) $$\;S_{fc}=2\pi \cdot r_{\mathrm{noz}}\cdot x+2\pi \cdot r_{\mathrm{noz}}\cdot R_{fp}\sum_{n=1}^{\infty }{\left[\frac{(2n-1)!!}{(2n)!!}\right]}^{2}\frac{{\left(\frac{r_{\mathrm{noz}}}{R_{fp}}\right)}^{2n}}{2n-1}$$

Thus, the average perpendicular distance $\;\hat{h}$ from the nozzle to the cylindrical flapper is as follows:

(6) $$\;\hat{h}=\frac{S_{fc}}{2\pi {\cdot r}_{noz}}$$

Next, the discharge characteristics of the cylindrical flapper will be discussed based on the calculated discharge area. As noted earlier, the closer the flow plane is to the geometric plane parallel to the cylindrical flapper axis, the greater the discharge resistance. Conversely, the closer the plane is to the one perpendicular to the axis, the lower the discharge resistance. From this, and based on the jet entrainment phenomenon [19], a hypothesis is proposed: air tends to flow preferentially toward the plane perpendicular to the cylindrical flapper axis, thereby facilitating discharge into the ambient environment.

**Figure 6:**
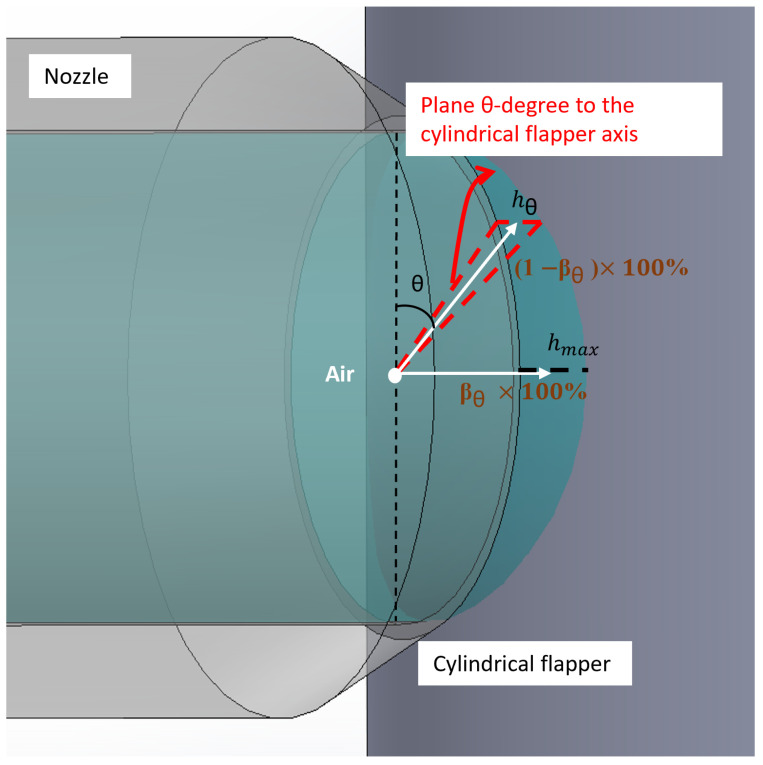
Schematic of assumed airflow tendency (view oriented at 35 degrees to the side view).

Assuming that the airflow induced by the rotation of the cylindrical flapper has a negligible effect on the nozzle discharge up to 20,000 rpm, which was proved in our previous results [5], and the mass percentage of air flowing toward the plane perpendicular to the cylindrical flapper axis (where $h_{\theta }$ = $h_{\max}$) accounts for a mass fraction $\beta_{\theta }$ × 100% of the total air discharged from the angular plane θ degree to the cylindrical flapper axis (as shown in Figure 6), and that air discharged through the angular plane θ degree to the cylindrical flapper axis (where $h_{\theta }$ = $h_{\theta }$) accounts for ($1-\beta_{\theta }$) × 100%, calculating the mass fraction across all angular planes yields the overall average mass fraction β:

(7) $$\beta =\frac{\sum \beta_{\theta }}{\theta }$$

The definition of β offers significant advantages in terms of both academic computation and industrial design. By homogenizing the irregular airflow domain through an averaged variable, it avoids the need for pointwise analysis of multiple irregular discharge areas while still achieving high computational accuracy. This approach does not rely on extensive experimental data or simulations, thereby greatly reducing both production and computational costs. Furthermore, in industrial applications, engineers can design nozzle–cylindrical flapper systems tailored to specific operating conditions without requiring deep specialized knowledge or advanced technical expertise, thus enabling a rapid response to the industrial demand for higher rotational speeds.

**Figure 7:**
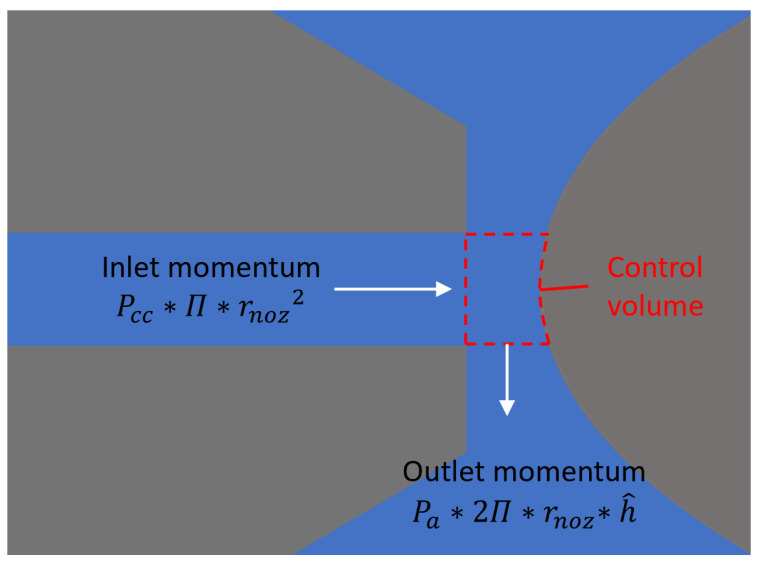
Analyzed control volume.

A control volume can be established as shown in Figure 7. The corresponding momentum integral equation is as follows:

(8) $$\begin{array}{c} \left(\frac{k}{k-1}\right)P_{cc}\cdot \pi {{\cdot r}_{noz}}^{2}=4\left(\frac{k}{k-1}\right)\cdot \int_{0}^{\frac{\pi }{2}}h_{\max}\cdot {\beta \cdot P}_{a}{\cdot r}_{noz}\cdot d\theta \\ +4\left(\frac{k}{k-1}\right)\cdot \int_{0}^{\frac{\pi }{2}}h_{\theta }\cdot {\left(1-\beta \right)\cdot P}_{a}{\cdot r}_{noz}\cdot d\theta \end{array}$$

where $P_{cc}$ represents the absolute control pressure of the nozzle–cylindrical flapper, $P_{a}$ represents atmospheric pressure, and *k* = 1.4 is the heat capacity ratio of air. Substituting Equations (2) and (3) into Equation (8), it becomes the following:

(9) $$\begin{array}{c} \left(\frac{k}{k-1}\right)P_{cc}\cdot \pi {{\cdot r}_{noz}}^{2}=\beta \cdot \left(\frac{k}{k-1}\right)\cdot P_{a}\cdot 2\pi {\cdot r}_{noz}\cdot h_{\max}\\ +\left(1-\beta \right)\cdot \left(\frac{k}{k-1}\right)\cdot P_{a}\cdot 2\pi {\cdot r}_{noz}\cdot \hat{h}=\left(\frac{k}{k-1}\right){\cdot S}_{fc}\cdot P_{a} \end{array}$$

For cases without the above flow tendency assumption (i.e., the traditional flat flapper case), the momentum integral equation is as follows:

(10) $$\begin{array}{c} \left(\frac{k}{k-1}\right)P_{cf}\cdot \pi {{\cdot r}_{noz}}^{2}=4\left(\frac{k}{k-1}\right)\cdot \int_{0}^{\frac{\pi }{2}}h_{\theta }{\cdot P}_{a}{\cdot r}_{noz}\cdot d\theta \end{array}$$

Substituting Equations (2) and (3) into Equation (10), it becomes the following:

(11) $$\begin{array}{c} \left(\frac{k}{k-1}\right)P_{cf}\cdot \pi {{\cdot r}_{noz}}^{2}=\left(\frac{k}{k-1}\right)\cdot P_{a}\cdot 2\pi {\cdot r}_{noz}\cdot \hat{h}\\ =\left(\frac{k}{k-1}\right){\cdot S}_{ff}\cdot P_{a} \end{array}$$

where $P_{cf}$ represents the absolute control pressure of the nozzle–flat flapper, and $S_{ff}$ represents the discharge area of the flat flapper. Based on these two momentum integral equations, under the same inlet (control pressure) and outlet (atmospheric pressure) conditions, the actual discharge area $S_{fc}$ of the cylindrical flapper with the assumed flow tendency should be greater than the discharge area $S_{ff}$ of the flat flapper without the assumed flow tendency. The corresponding relationship coefficients $\eta_{c}$ are as follows:

(12) $$\begin{array}{c} \eta_{c}=\frac{S_{fc}}{S_{ff}}=\beta \frac{h_{\max}}{\hat{h}}+\left(1-\beta \right) \end{array}$$

## 4. Calibrated Theoretical Calculation Model of Nozzle Cylindrical Flapper System

### 4.1. Establishment of Calibrated Model

The theoretical calculation model for the traditional nozzle–flat flapper developed by Colin [18] is introduced here. Based on this model and the assumptions proposed in this study, a calibrated theoretical model for the nozzle–cylindrical flapper system is developed and numerically computed.

Colin’s model employs spatial integration methods based on the following continuity equation:

(13) $$\begin{array}{c} {\;\int \int \int \;}_{D}div\left(\rho V\right)\;dD=0 \end{array}$$

where ρ represents the density of air, *V* is the air velocity, and *D* represents the volume. This is combined with the momentum conservation equation:

(14) $$\begin{array}{c} {\;\int \int \int \;}_{D}div\left(\rho V⊗V+PI-\sum v\right)\;dD=0 \end{array}$$

where *P* represents the absolute pressure, *I* is the unit tensor, and *v* represents the perpendicular velocity. The total enthalpy equation is expressed as follows:

(15) $$\begin{array}{c} {\;\int \int \int \;}_{D}div\left(\rho Vh_{i}-\sum v\cdot V-\lambda gradT\right)\;dD=0 \end{array}$$

where $h_{i}$ represents stagnation enthalpy, λ is the thermal conduction coefficient, and *T* represents the air temperature. To simplify these equations, the compressible Bernoulli equation with Mach number *M* is used:

(16) $$\begin{array}{c} \;\frac{P}{P_{i}}=\omega \left(M\right)={\left(1+\frac{k-1}{2}M^{2}\right)}^{-\frac{k}{k-1}}\\ \frac{T}{T_{i}}=\theta \left(M\right)={\left(1+\frac{k-1}{2}M^{2}\right)}^{-1} \end{array}$$

with an additional function to define the Mach number:

(17) $$\begin{array}{c} \sigma \left(M\right)=1+kM^{2} \end{array}$$

This results in three sets of implicit iterative equations that describe the flow domain interactions among the various components of the nozzle–flapper system.

First, the coupling equations between the orifice and the control room are obtained:

(18) $$\begin{array}{c} \frac{\sigma \left(M_{c}\right)\sqrt{\theta (M_{c})}}{M_{c}}=\frac{\sigma \left(M_{o}\right)\sqrt{\theta (M_{o})}}{M_{o}}\left[1+\frac{\frac{S_{c}}{S_{o}}-1}{\sigma (M_{o})}\right] \end{array}$$

where $M_{c}$ is the Mach number in the control room, and $M_{o}$ is the Mach number in the orifice. $S_{c}$ and $S_{o}$ are the cross-sectional areas of the control room and orifice, respectively.

When $M_{o}$ > 1, this is replaced by the following:

(19) $$\begin{array}{c} \;M_{o}=1 \end{array}$$

Next, the coupling equation between the control room and the nozzle is obtained:

(20) $$\begin{array}{c} \frac{\omega (M_{c})}{\sqrt{\theta (M_{c})}}M_{c}\frac{S_{c}}{S_{f}}=\frac{\omega (M_{f})}{\sqrt{\theta (M_{f})}}M_{f} \end{array}$$

where $M_{f}$ is the Mach number in the discharge area between the nozzle and the flapper, and $S_{f}$ is the corresponding cross-sectional area.

Finally, the coupling equations between the orifice and the nozzle are derived:

(21) $$\begin{array}{c} \frac{\sqrt{\theta (M_{f})}}{\omega (M_{f})}=\frac{\sqrt{\theta (M_{o})}}{\omega \left(M_{o}\right)M_{o}}\frac{S_{f}}{S_{o}}\frac{P_{a}}{pi_{o}} \end{array}$$

When $M_{f}$ > 1, this is replaced by the following:

(22) $$\begin{array}{c} M_{f}=1 \end{array}$$

The calibration method proposed in this study is then applied. Since the focus is on the linear region where the control pressure varies linearly with the distance from the nozzle to the flapper, the derived coefficient can be substituted into Equations (20) and (21):

(23) $$\Delta S_{fc}=\eta_{c}\Delta S_{f}$$

The initial control pressure $P_{ci}$ is defined based on the experimental results at *x* = 0. The assumed mass fraction β is set to 0%, 25%, 50%, 75%, and 100%. Based on these assumptions, numerical calculations are performed.

**Figure 8:**
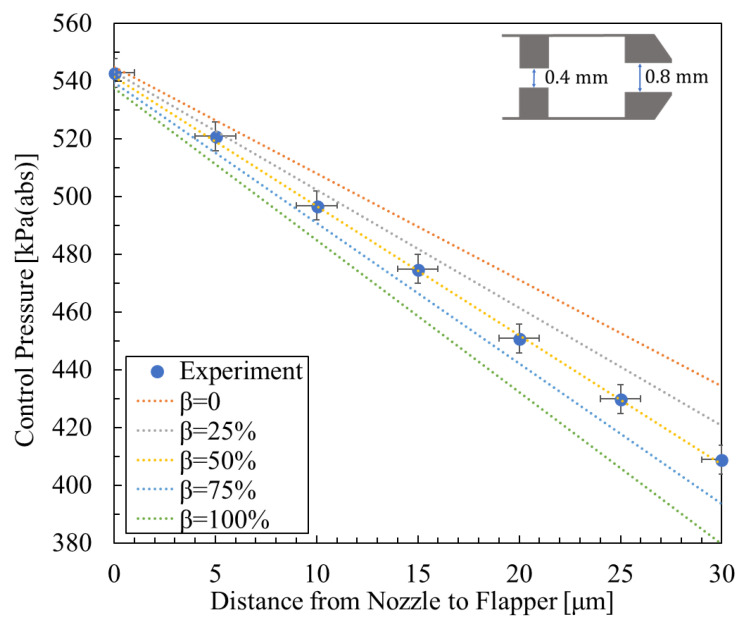
Experimental result [5] and calculated results for the relationship between control pressure and nozzle-to-flapper distance, with nozzle diameter 0.8 mm, orifice diameter 0.4 mm, and supply pressure 600 kPa (abs).

**Figure 9:**
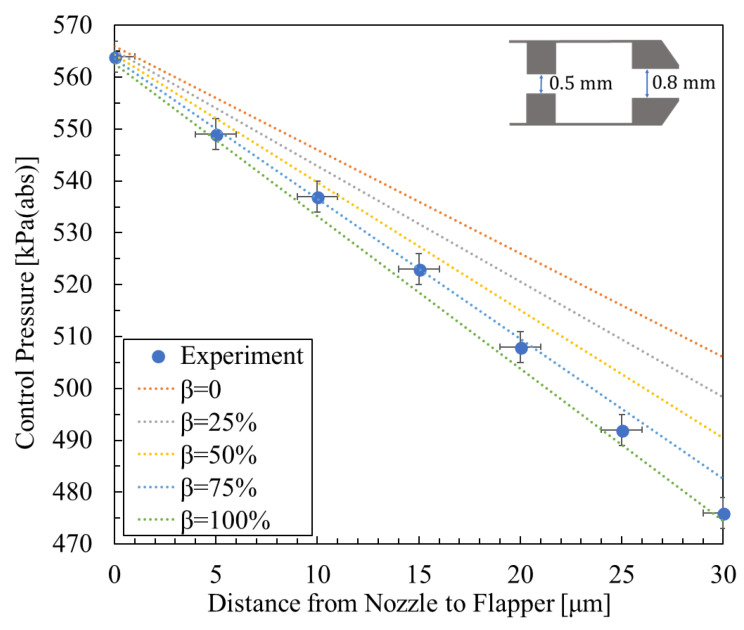
Experimental result [5] and calculated results with nozzle diameter 0.8 mm, orifice diameter 0.5 mm, and supply pressure 600 kPa (abs).

**Figure 10:**
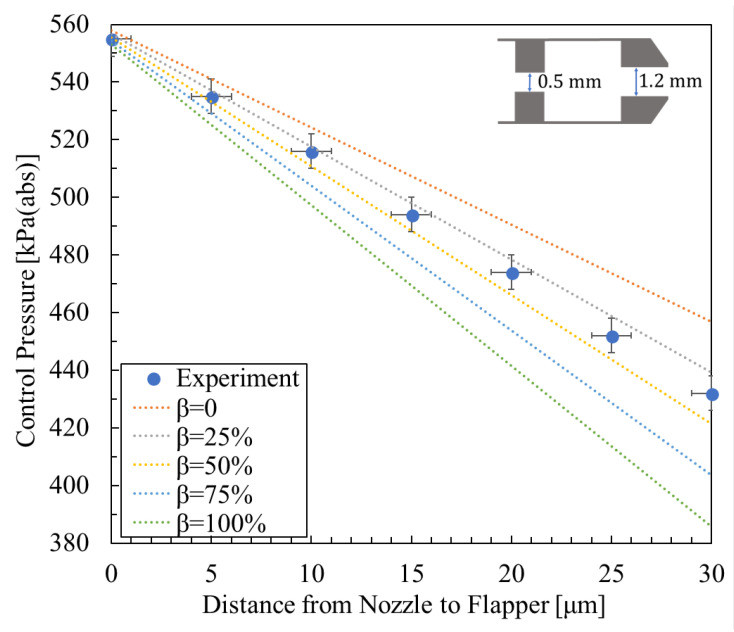
Experimental result [5] and calculated results with nozzle diameter 1.2 mm, orifice diameter 0.5 mm, and supply pressure 600 kPa (abs).

For the experimental data cited below, as well as for the additional validation data, the methods of error analysis and calibration repeatability were established as follows: First, the measurement uncertainty of the Distance Regulator (LD-243-C7) is specified as ±1 μm. The pressure sensor (AP-13S) has a measurement uncertainty defined as a full-scale measurement error of ±0.5% of F.S. Accordingly, in Figure 8, Figure 9 and Figure 10, the error bars are defined as follows: the horizontal displacement error is taken as ±1 μm, while the vertical pressure error is defined as the pressure error induced by the ±1 μm displacement error, combined with a pressure sensor measurement error of ±0.5% of F.S.

The repeatability of the experimental measurements was evaluated over three independent trials. The measured displacement values exhibited a repeatability error of less than 6.7%, while the measured control pressure exhibited a repeatability error of less than 7.2%.

Figure 8, Figure 9 and Figure 10 show the relationship between control pressure and the nozzle-to-flapper distance for the actual nozzle–cylindrical flapper system [5] compared with the theoretically calculated results for various orifice and nozzle combinations. Structural dimensions and supply pressures are specified in the figures.

From the comparisons, it can be concluded that, compared to the nozzle–flat flapper system (corresponding to β = 0 in the figures), the static characteristics of the nozzle–cylindrical flapper system, i.e., its sensitivity to control pressure variation, are higher. This confirms that the cylindrical flapper system exhibits lower discharge resistance.

Furthermore, the impact of the assumed airflow tendency becomes more pronounced when the nozzle and orifice diameters are more similar. For example, in Figure 9 ($r_{noz}$ = 0.8 mm, and $r_{ori}$ = 0.5 mm), the best match to the experimental data corresponds to β = 100%. In contrast, when the nozzle and orifice sizes differ more significantly, as in Figure 8 and Figure 10, the best-matching results correspond to β = 50% and β = 25%, respectively. This trend can be explained as follows: for the same nozzle-to-flapper distance, when the orifice diameter decreases or the nozzle diameter increases, the relative similarity in size reduces the flow between the nozzle and flapper. As a result, the viscous resistance along the various angular planes of discharge becomes smaller, making it easier for airflow to discharge along these paths. Therefore, the influence of the assumed flow tendency diminishes, corresponding to a smaller β value.

### 4.2. Estimation and Validation of the Calibrated Model

Based on the results from the Section 4.1, it can be concluded that the static characteristics of the nozzle–cylindrical flapper system differ from those predicted by existing theoretical models. By introducing the airflow assumption between the nozzle and flapper proposed in this study, a compensation coefficient is added to the existing models, enabling theoretical results that more closely match the actual static behavior of the nozzle cylindrical flapper system under different assumed flow mass fractions β × 100%. To achieve more accurate computational results, further investigation is required into the relationship between system dimensions and the corresponding mass fraction β × 100%.

In the Section 3, the influence of the cylindrical flapper’s radius on the discharge area was incorporated into the flow assumption. Therefore, the current discussion focuses on the orifice and nozzle diameters. To generalize combinations of orifice and nozzle diameters, a dimensionless ratio ϕ between the orifice diameter $d_{ori}$ and nozzle diameter $d_{noz}$ is introduced and defined as follows:

(24) $$\phi =\frac{d_{ori}}{d_{noz}}$$

An advantage of this definition is that, for the nozzle flapper system to function properly, the nozzle diameter must be larger than the orifice diameter [20,21]. Thus, the dimensionless ratio ϕ is constrained between 0 and 1, facilitating the generalization and analysis of the results.

Additionally, the required working distance for the nozzle flapper corresponding to the aerostatic spindle in this study is 30 μm. From previous results [5], it was observed that when ϕ < 0.4, the effective working distance of the nozzle cylindrical flapper system is less than 30 μm. Therefore, to develop a theoretical model suitable for the aerostatic spindle in this study, only data with ϕ > 0.4, as provided in Figure 8, Figure 9 and Figure 10, are used to analyze and fit the assumed flow mass fractions β.

It should be noted that the modeling approach proposed in this study requires the simultaneous fulfillment of two conditions: (i) the rotational effects can be neglected, and (ii) within the range of 0–30 μm the control pressure exhibits a linear relationship with displacement. If the rotational effects cannot be neglected, the model can no longer be analyzed under the assumption of axial symmetry, and the discharge characteristics of the gas in the plane perpendicular to the cylindrical flapper axis will be significantly affected. Similarly, if the linear relationship between control pressure and displacement does not hold within the range of 0–30 μm—that is, when the flow rate between the nozzle and the cylindrical flapper is too small, such that the compressed gas does not fully occupy the entire clearance region—the actual flow behavior will deviate considerably from the assumed flow tendency. Therefore, in order to ensure the accuracy of the correction coefficient proposed in this study, it is essential that both of the above conditions are satisfied simultaneously. The specific methodology is as follows.

**Figure 11:**
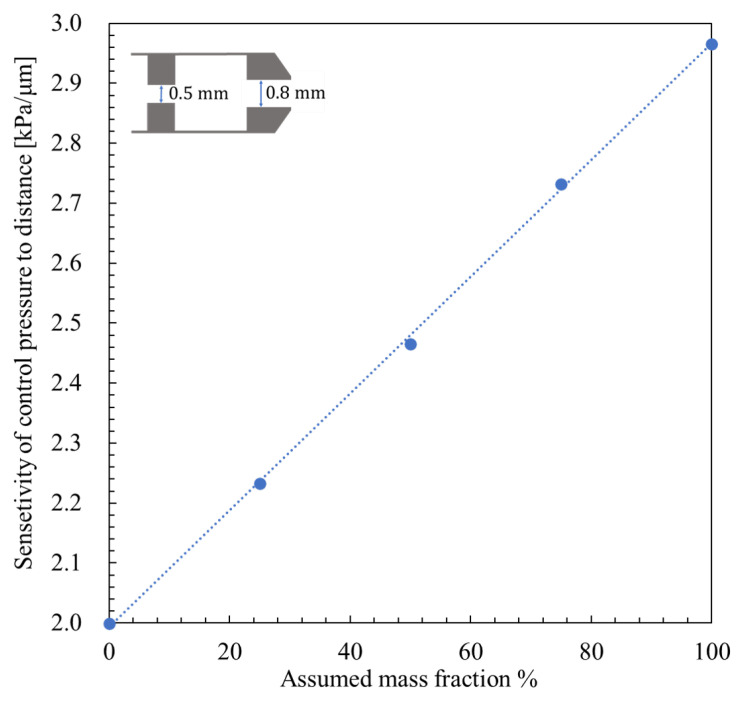
Sensitivity of control pressure to distance and mass fraction β with fitted lines (nozzle diameter 0.8 mm, orifice diameter 0.5 mm, supply pressure 600 kPa abs).

First, as shown in Figure 11, under a supply pressure of 600 kPa (abs), for a nozzle diameter $d_{noz}$ = 0.8 mm and orifice diameter $d_{ori}$ = 0.5 mm, the sensitivity of control pressure to displacement was calculated for nozzle to flapper distances ranging from 0 to 30 μm, using *β* values of 0%, 25%, 75%, and 100%. The resulting sensitivity data were linearly fitted with respect to *β*, and by substituting the experimentally obtained sensitivity, the corresponding *β* values were determined. This method was also applied to combinations with a nozzle diameter of 0.8 mm and orifice diameter of 0.4 mm, and a nozzle diameter of 1.2 mm with an orifice diameter of 0.5 mm. The results are presented in Table 1.

Next, the relationship between the dimensionless ratio and the mass fraction was fitted. The sigmoid shape observed in the variation in the mass fraction *β* with respect to the dimensionless ratio *ϕ* is not merely a mathematical artifact but instead has a clear physical basis related to the flow characteristics of the nozzle–cylindrical flapper system. The sigmoid curve can be divided into three distinct regions, each corresponding to a different flow regime depending on the ratio between the orifice and nozzle diameters.

Region I: Low Dimensionless Ratio (ϕ<0.4). In this range, the orifice diameter is significantly smaller than that of the nozzle, resulting in a relatively low overall airflow rate. The discharge area between the nozzle and the cylindrical flapper is relatively unobstructed, and the pressure distribution is more uniform across all angular faces. As a result, the effect of the proposed flow hypothesis—based on the varying effective discharge area—is minimal. This leads to a plateau in *β* values, representing the lower asymptote of the sigmoid curve.

Region II: Moderate Dimensionless Ratio (0.4<ϕ<0.8). Here, the orifice diameter reaches an optimal proportion relative to the nozzle. The total airflow increases, and the effective discharge area between the nozzle and the flapper becomes more sensitive to changes in clearance. This is the region where the effect of our flow hypothesis becomes most significant, leading to a rapid increase in *β*. This corresponds to the steep middle section of the sigmoid curve.

Region III: High Dimensionless Ratio (ϕ>0.8). In this range, the orifice diameter becomes too large relative to the nozzle. Similar to Region I, the overall discharge is now dominated by the physical size limitation of the nozzle itself, rather than the clearance-dependent effective area. As a result, further increases in *ϕ* no longer significantly enhance the influence of the flow hypothesis, and *β* tends to plateau again, forming the upper asymptote of the sigmoid curve.

In conclusion, while the use of a sigmoid function may initially appear empirical, its behavior is consistent with the underlying physical mechanisms of the system. Thus, a generalized sigmoid function was used for fitting the data in Table 1 to better reflect these constraints and improve accuracy.

To validate the fitted curve, experiments were conducted under the same supply pressure of 600 kPa (abs) using the following test cases: nozzle diameter of 0.8 mm and orifice diameter of 0.6 mm, nozzle diameter of 1.2 mm and orifice diameter of 0.6 mm, nozzle diameter of 1.2 mm and orifice diameter of 0.7 mm, and nozzle diameter of 1.2 mm and orifice diameter of 0.8 mm. Using the same procedure as the original datasets, the corresponding dimensionless ratios and mass fractions were obtained, as shown in Table 2.

**Figure 12:**
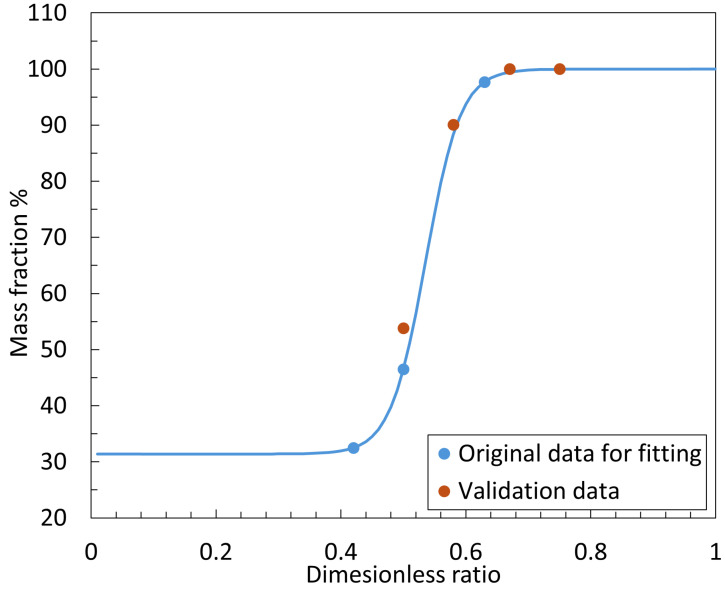
Fitted function and experimental data of dimensionless ratio ϕ to mass fraction β.

As shown in Figure 12, the original experimental data (blue dots) and the generalized sigmoid fitting curve (blue line) are compared with the validation (orange dots). It can be observed that the fitted function aligns well with the validation data. Therefore, this fitted function can be used to predict the static characteristics of the nozzle cylindrical flapper system within a working distance of 0–30 μm and for dimensionless ratios ranging from 0.4 to 1 under a supply pressure of 600 kPa (abs). The specific formula for the fitted function is as follows:

(25) $$\;\beta =[\frac{68.61}{1+\exp\left(-35.58\cdot \phi +19.06\right)}+31.39]\times 100\%$$

To ensure the general applicability of the proposed method, a model under a supply pressure condition of 700 kPa (abs) was also calculated following the same research procedure. Using the identical experimental system, procedure, and combinations of orifice and nozzle diameters, the experiments for model fitting, as shown in Table 3, were carried out.

Accordingly, the fitted equation under the supply pressure condition of 700 kPa (abs) was obtained:

(26) $$\;\beta =[\frac{75.96}{1+\exp\left(-22.80\cdot \phi +12.75\right)}+24.04]\times 100\%$$

Based on the above data, the sigmoid model fitting was conducted. Similarly, to verify the stability and accuracy of the model predictions under the supply pressure condition of 700 kPa (abs), validation experiments were also carried out in parallel using the data shown in Table 4.

**Figure 13:**
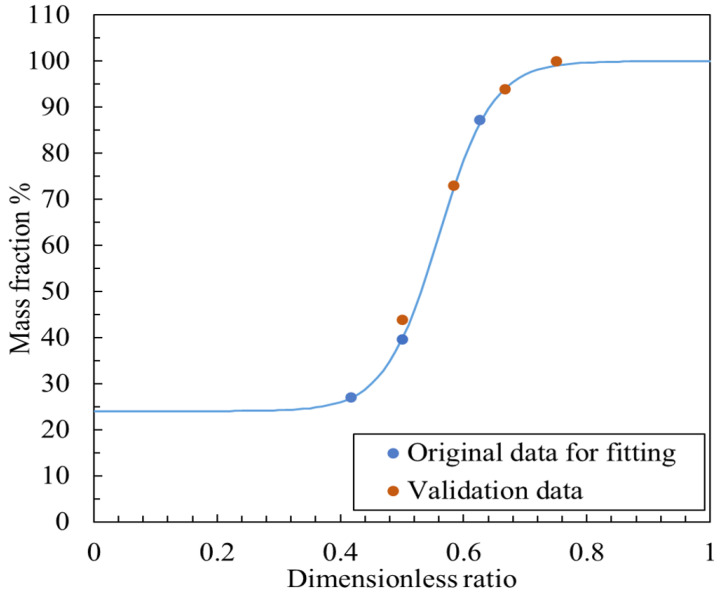
Fitted function and experimental data of dimensionless ratio ϕ to mass fraction β under 700 kPa (abs) supply pressure.

In conjunction with the above validation experimental results, the model under this supply pressure condition and its corresponding validation outcomes are presented in Figure 13. This model likewise exhibits high computational accuracy and reliability, comparable to that of the model at a supply pressure of 600 kPa (abs).

## 5. Conclusions

In this paper, a theoretical model was developed to predict and describe the static characteristics of the nozzle–cylindrical flapper system. Building upon previous research, this study investigated the unique features of the discharge area in the nozzle–cylindrical flapper configuration. By comparing the discharge characteristics of planar and curved surfaces, the differences between the nozzle–cylindrical flapper system and the traditional nozzle–flat flapper system were highlighted. It was concluded that the nozzle–cylindrical flapper system exhibits lower discharge resistance. Through the calculation of the discharge area, a flow assumption for air discharge was proposed, using mass fraction as its foundation. This assumption was then applied to derive the momentum equation for the nozzle–cylindrical flapper system, from which a momentum correction coefficient was obtained. By incorporating this coefficient, the existing Colin model was calibrated, resulting in an implicit mathematical model suitable for calculating the static characteristics of the nozzle–cylindrical flapper system. The model was further refined using existing data to improve its accuracy, and its predictive performance was validated through experimental verification.

Although the model developed in this study has certain limitations—for instance, due to the practical requirements of the industrial application under consideration, the analysis and modeling were limited to a cylindrical flapper with a diameter of 16 mm, and potential variations in ambient pressure may arise when the nozzle–flapper system is subsequently integrated with the air spindle—two points should be emphasized. First, the most significant contribution of the theoretical model is that it opens up the possibility of applying nozzle–flapper systems to distance measurement in high-speed rotating bodies, thereby providing researchers and engineers with a methodological basis for customized design according to their specific operational requirements. Second, future research may further refine the present model by adopting a similar approach that incorporates experimental data to achieve closer alignment with actual working conditions.

## Figures and Tables

**Table 1:** Dimensionless ratio and mass fraction results.

Dimensionless Ratio ϕ	Mass Fraction β × 100%
0.5/1.2 = 0.42	32.5
0.4/0.8 = 0.50	46.5
0.5/0.8 = 0.63	97.7

**Table 2:** Validation experimental results of dimensionless ratio and mass fraction.

Dimensionless Ratio ϕ	Mass Fraction β × 100%
0.6/0.8 = 0.75	100
0.6/1.2 = 0.50	53.8
0.7/1.2 = 0.58	90.1
0.8/1.2 = 0.67	100

**Table 3:** Dimensionless ratio and mass fraction results under 700 kPa (abs) supply pressure.

Dimensionless Ratio ϕ	Mass Fraction β × 100%
0.5/1.2 = 0.42	27.1
0.4/0.8 = 0.50	39.7
0.5/0.8 = 0.63	87.4

**Table 4:** Validation experimental results of dimensionless ratio and mass fraction under 700 kPa (abs) supply pressure.

Dimensionless Ratio ϕ	Mass Fraction β × 100%
0.6/0.8 = 0.75	100
0.6/1.2 = 0.50	44
0.7/1.2 = 0.58	73.1
0.8/1.2 = 0.67	94.1

## Data Availability

Data are contained within the article.

## Abbreviations

β	Mass fraction of air discharged toward the perpendicular plane [–]
ϕ	Dimensionless ratio of orifice diameter to nozzle diameter, $d_{ori}/d_{noz}$ [–]
$d_{ori}$	Orifice diameter [mm]
$d_{noz}$	Nozzle diameter [mm]
$R_{fp}$	Radius of cylindrical flapper [mm]
$r_{noz}$	Nozzle radius [mm]
$r_{\theta }$	Projected radius at circumferential angle θ [mm]
$h_{\theta }$	Perpendicular distance between nozzle and flapper at angle θ [µm]
$h_{max}$	Maximum perpendicular distance between nozzle and flapper [µm]
$\hat{h}$	Average perpendicular distance between nozzle and flapper [µm]
$S_{fc}$	Effective discharge area of cylindrical flapper [mm^2^]
$S_{ff}$	Effective discharge area of flat flapper [mm^2^]
$\eta_{c}$	Compensation coefficient for cylindrical flapper [–]
$P_{cc}$	Absolute control pressure of nozzle–cylindrical flapper [Pa]
$P_{cf}$	Absolute control pressure of nozzle–flat flapper [Pa]
$P_{a}$	Ambient (atmospheric) pressure [Pa]
*k*	Heat capacity ratio of air (typically 1.4) [–]
ρ	Density of air [kg/m^3^]
*V*	Air velocity vector [m/s]
*M*	Mach number [–]
$M_{c}$	Mach number in the control room [–]
$M_{o}$	Mach number at the orifice [–]
$M_{f}$	Mach number at the nozzle–flapper discharge region [–]
$S_{c}$	Cross-sectional area of control room [mm^2^]
$S_{o}$	Cross-sectional area of orifice [mm^2^]
$S_{f}$	Cross-sectional discharge area between nozzle and flapper [mm^2^]
$P_{i}$	Inlet (supply) pressure [Pa]
*T*	Air temperature [K]
$h_{i}$	Stagnation enthalpy [J/kg]
λ	Thermal conduction coefficient [W/(m·K)]
$\Delta S_{fc}$	Corrected discharge area increment for cylindrical flapper [mm^2^]
